# After-work recovery in hybrid work: remote work intensity and general self-efficacy in work-related rumination

**DOI:** 10.3389/fpsyg.2026.1845951

**Published:** 2026-07-28

**Authors:** Emma Velati, Simone Donati, Serena Rubini, Simona Margheritti, Andrea Gragnano, Paola Gatti, Tatiana Kochetova, Andrea Ceschi, Salvatore Zappalà, Massimo Miglioretti

**Affiliations:** 1 Department of Psychology, University of Milano-Bicocca, Milan, Italy; 2 Department of Law, University of Milano-Bicocca, Milan, Italy; 3 Department of Psychology, University of Bologna, Bologna, Italy; 4 Department of Human Sciences, University of Verona, Verona, Italy

**Keywords:** affective rumination, general self-efficacy, hybrid work, problem-solving pondering, work recovery

## Abstract

**Introduction:**

Hybrid work is increasingly widespread; however, it remains unclear whether employees benefit from these arrangements or struggle to manage work–life boundaries and achieve recovery. Focusing on two early cognitive indicators of recovery, affective rumination (AffRum) and problem-solving pondering (PSP), this study examines whether general self-efficacy (GSE), as a foundational personal resource, shapes the relationship between remote work intensity (RWI; operationalized as the structural frequency of remote workdays) and these processes. In high-intensity hybrid settings, where reduced institutional scaffolding and blurred boundaries amplify demands for autonomous boundary management, such personal resources become crucial.

**Methods:**

Data were collected from 747 hybrid employees from four medium-to-large Italian organizations.

**Results:**

Results showed that RWI interacted with GSE in predicting AffRum, such that high RWI was associated with higher rumination among employees with low GSE. No such interaction was found for PSP; suggesting that work-related problem-solving warrants further investigation to determine whether it stems from its constructive problem-solving nature or from how the construct is operationalized and perceived. Overall, our correlational findings suggest that intensive remote work configurations may present specific risks for recovery when personal self-regulatory resources are limited.

**Discussion:**

Rather than focusing solely on the structural number of remote days, hybrid work management could benefit from incorporating strategies that support employees’ boundary management and psychological disengagement capabilities.

## Introduction

Hybrid work has become increasingly common, reshaping how employees manage their work lives (Gratton, 2021; Kniffin et al., 2021). However, it remains unclear whether employees who engage in these arrangements actually benefit from them or instead experience difficulties in managing work-life boundaries and achieving adequate recovery (Demerouti et al., 2014; Allen et al., 2015). While COVID-19 accelerated hybrid work, its post-pandemic adoption has stabilized and continues to evolve (Gratton, 2021; Kniffin et al., 2021; Miglioretti, 2023). However, hybrid practices do not inherently guarantee flexibility or effective recovery. Various remote work terms are often used interchangeably, despite providing different levels of support for well-being. For instance, while ‘remote work’ typically refers to work performed off-site, ‘mobile work’ specifically emphasizes its itinerant nature (Hislop and Axtell, 2009). Conversely, flexible work entails a broader combination of spatial, temporal, and organizational flexibility (Timms et al., 2015; Spreitzer et al., 2017). While hybrid work is increasingly widespread, its various applications do not necessarily align with the principles of agile work (Miglioretti et al., 2021). For instance, organizations typically attempt to optimize remote work arrangements and their impact on off-job time by adjusting the number of days spent working off-site, thereby increasing or decreasing remote work intensity (Belle et al., 2015; Heiden et al., 2021; Margheritti et al., 2023). Furthermore, there is a growing trend toward decreasing intensity (i.e., number) of remote workdays, scaling back the frequency of remote work (Bloom et al., 2024). While essential, this metric is insufficient to guarantee hybrid work quality. Contractual shifts in remote days do not inherently enhance perceived flexibility (Miglioretti et al., 2021) if they fail to alter the cognitive and temporal boundaries shaping post-work life. Indeed, many regulatory frameworks emphasize spatial flexibility at the expense of temporal autonomy and psychological detachment (Miglioretti et al., 2021). Despite increased flexibility, the ability of hybrid employees to effectively disengage and recover remains uncertain. Since organizations continue to prioritize remote work frequency as their primary metric, even though it does not guarantee work quality, investigating these intensity dynamics is crucial.

From a recovery perspective (Derks et al., 2015), what matters is not only *where* or *how often* (hybrid) work is performed, but whether work arrangements allow employees to disengage cognitively from work demands during non-work time. For example, previous studies indicate that hybrid work is often associated with cognitive pre-occupation during non-work hours, especially where high autonomy lacks the support of effective boundary management strategies (Allen et al., 2015; Derks et al., 2015; Sonnentag, 2018).

Given these premises, research must clarify how organizations can leverage hybrid practices to support well-being via recovery. As boundaries blur, constant connectivity in remote work may hinder recovery by fostering both affective rumination and problem-solving pondering (Cropley et al., 2023). We consider affective rumination as a form of intrusive, repetitive, and emotionally charged work-related rumination that leads to recurrent work-related thoughts during off-job time, accompanied by emotional distress and problem-solving pondering as a form of repetitive cognitive work-related rumination that also occurs during off-job periods and is oriented toward problem resolution (Cropley and Zijlstra, 2011; Cropley et al., 2023). The literature has consistently shown that insufficient recovery from work can have detrimental effects on employees’ health and well-being. Recovery failure unfolds progressively, beginning at the cognitive level before emotional and physical strain emerge (McEwen, 1998; Eden, 2001). The quality of the recovery experience, and not merely the amount of time available for recovery, plays a crucial role in the process (Etzion et al., 1998). Within the recovery framework, a high level of affective rumination can lead to complications in work-home management (Junker et al., 2021). Recovery failure begins not with obvious exhaustion or reduced well-being, but with continued work-related thoughts after working hours (Brosschot et al., 2006; Sonnentag and Fritz, 2015). Affective rumination and problem-solving pondering, therefore, represent the initial cognitive stage in the process of post-work adjustment failure. For this reason, these dimensions are increasingly seen as proximal outcomes of job exposure, occurring before affective, behavioral, and health outcomes (Vahle-Hinz et al., 2017; Weigelt et al., 2019).

Previous literature has yet to elucidate the impact of remote work intensity on recovery, recognizing personal and individual resources as crucial drivers of these experiences (Sonnentag and Fritz, 2007). This is particularly relevant as remote work increases demands for autonomy and self-regulation, making recovery more dependent on an individual’s ability to manage boundaries and disengage. Consequently, personal resources like self-efficacy become crucial. While self-efficacy is known to buffer stress and support recovery, its specific interaction with remote work frequency in shaping cognitive recovery remains unclear. To address this gap, the present study focuses on remote work intensity (i.e., the number of remote workdays) as a key structural condition and examines its association with proximal cognitive recovery processes (i.e., affective rumination and problem-solving pondering), while considering the role of self-efficacy in shaping these relationships.

## Theoretical background

### Remote work intensity

Remote work often involves greater delegation of responsibility and self-directed goal management (Contreras et al., 2020). However, flexibility and autonomy are not necessarily achieved simply by implementing a formal hybrid-work policy (Kossek et al., 2006; Delanoeije and Verbruggen, 2020; Gajendran et al., 2024). When hybrid work increases discretion over time and space without adequate boundary support, it may foster “constant connectivity,” a pattern of heightened availability demands and continued ICT-mediated coordination that can extend beyond scheduled hours (Mazmanian et al., 2013).

Importantly, remote work intensity (i.e., the frequency of remote working days) should also be understood as a structural work condition that is theoretically and operationally distinct from autonomy. At the same time, RWI can be understood as a structural work arrangement that may increase employees’ exposure to autonomy-related working conditions: employees who work remotely more frequently are, on average, more likely to be operating in conditions that require self-directed goal management, independent scheduling, and reduced institutional scaffolding (Contreras et al., 2020; Donati et al., 2025). In this interpretive stance, high-RWI conditions may activate autonomy-related processes.

Digital work intensity affects employees differently based on their resources. Crucially, higher intensity often correlates with poorer working-time quality, despite simultaneous gains in skill and engagement (Rodríguez-Modroño, 2022). Similarly, intensity shows non-linear associations with work engagement, with low to moderate levels tending to be more favourable than very high intensity (Nagata et al., 2021). Evidence from hybrid workers indicates that work-time autonomy is associated with remote work intensity. This indicates that intensity may function as a resource-driven choice rather than a traditional demand-driven stressor (Donati et al., 2025). This pattern is consistent with the broader “too-much-of-a-good-thing” logic whereby the benefits of an initially positive work condition diminish and eventually reverse beyond a certain threshold (Zhou, 2020). This heterogeneity in outcomes as a function of individual resources motivates the present study’s focus on general self-efficacy as a boundary condition, rather than assuming a uniform directional effect of RWI on recovery cognitions, it would be fundamental to test whether the relationship varies across levels of this personal resource.

High RWI often impairs well-being by heightening expectations of ICT responsiveness. This surge in daily virtual communication limits opportunities for disengagement and prevents effective evening relaxation (Parker et al., 2026). A second pathway involves blurred work-life boundaries: as remote work frequency increases, more frequent boundary management episodes and greater ‘boundary work’ may reduce the likelihood of psychological detachment. A third, day-level pathway concerns the depletion of recovery-supportive conditions; remote days often entail fewer breaks and lower perceived inclusion, which can drain daily energy and promote poorer evening recovery (Parker et al., 2026). These mechanisms align with recovery perspectives emphasizing the need for reduced work-related activation and effective detachment during off-job time (Zijlstra and Sonnentag, 2006) and post-work recovery (Eden, 2001; Cropley et al., 2023).

### Work-related rumination in the recovery process

Work recovery refers to the process through which individuals restore their psychophysical resources after prolonged periods of work effort (Cropley and Zijlstra, 2011). Persistent work-related thinking is a major barrier to recovery. As an umbrella term, rumination encompasses distinct states: specifically affective rumination refers to intrusive, repetitive, and emotionally negative work-related thoughts that are typically accompanied by heightened activation, and is therefore considered a maladaptive recovery-related cognition. In contrast, problem-solving pondering refers to repetitive but solution-focused thinking aimed at addressing work issues; it is often less detrimental and may even be experienced as functional, although sustained problem-focused thinking can still undermine recovery if repetitive and prolonged (Cropley et al., 2012; Querstret and Cropley, 2012).

Within recovery research, impaired recovery is commonly conceptualized as developing over time, with sustained cognitive activation preceding downstream emotional and physiological strain (McEwen, 1998; Eden, 2001).

Accordingly, and consistent with Cropley et al. (2012), we focused on two cognitive components of recovery-related rumination, affective rumination and problem-solving pondering, rather than including all recovery dimensions (i.e., relaxation). Therefore, these cognition-focused indicators capture how work experiences “carry over” into leisure time by indexing what remains mentally active before more distal outcomes (i.e., strain reactions or health complaints) are detectable. From this perspective, affective rumination reflects a maladaptive state of after-hours activation; conversely, problem-solving pondering represents a more functional cognitive mode that nonetheless hinders detachment when it becomes chronic.

However, reflecting on a work-related issue does not necessarily lead to negative consequences. Analyzing a specific problem or evaluating one’s work activity with the aim of identifying potential improvements may also be advantageous (Cropley and Zijlstra, 2011). In line with research linking certain forms of reflective, solutions-oriented thinking to creativity and innovation (Baas et al., 2008; Spoor et al., 2010), problem-solving pondering may be beneficial, including by fostering actionable plans to address work issues even when the problem is not fully resolved (Syrek et al., 2017). Hence, thinking about work during off-work time is not inherently harmful. Considering this, problem-solving pondering is often framed as goal-directed thinking (Kinnunen et al., 2017). Nevertheless, problem-solving pondering may also increase under conditions of greater connectivity and, although often framed as more functional, could carry costs for health over time (Querstret and Cropley, 2012).

Taken together, the three pathways described above, telepressure, blurred boundaries, and depletion of recovery-supportive conditions, each constitute theoretically distinct but converging mechanisms through which increasing RWI would be expected to elevate work-related cognitive activation after hours. Because affective rumination is specifically triggered by emotionally laden, unresolved work experiences (Cropley et al., 2012), and because higher RWI structurally increases exposure to such experiences by extending work-related ICT contact into off-job time (Barber and Santuzzi, 2015), a positive association between RWI and AffRum represents the theoretically most direct prediction. The same logic, albeit less strongly due to his more functional frame, applies to PSP: greater connectivity implies more unfinished or pending tasks entering non-work hours, which may sustain solution-focused thinking (Kinnunen et al., 2017; Syrek et al., 2017). On the basis of these converging arguments, we expected higher RWI to be positively associated with both recovery-related cognitions. We then hypothesize the following:

*H1*: Remote work intensity is positively associated with greater affective rumination (H1a) and greater problem-solving pondering (H1b) as distinct cognitive responses to work.

### Self-efficacy as a resource

Self-efficacy is people’s belief in their ability to organize and execute the courses of action required to manage future situations and attain specific levels of performance (Rumsey et al., 2013; Waddington, 2023). It reflects an optimistic belief in one’s ability to cope with stressful or difficult situations and can guide behavioral change and support optimal functioning (Schwarzer and Hallum, 2008). Self-efficacy is linked to cognitive, motivational, and affective processes (Rumsey et al., 2013).

The post-work recovery literature suggests that self-efficacy can function as a personal resource that facilitates recovery processes. This aligns with Conservation of Resources Theory (Hobfoll, 2011), which holds that stress occurs when resources are threatened or lost, while recovery requires their replenishment. Consequently, individuals with high self-efficacy feel more capable of managing job demands and establishing effective work-life boundaries (Sonnentag and Fritz, 2007). More directly, Sonnentag and Kruel (2006) showed that recovery-related self-efficacy is a key antecedent of psychological detachment, a finding validated by both self and family reports. Belief in one’s recovery potential is essential in hyperconnected environments, as it helps protect against the resource depletion caused by blurred boundaries and the pressure for constant availability (Rodríguez-Modroño, 2022). Likewise, employees with higher self-efficacy cope more effectively with job stressors, supporting faster recovery processes (Demerouti et al., 2012).

**Figure 1:**
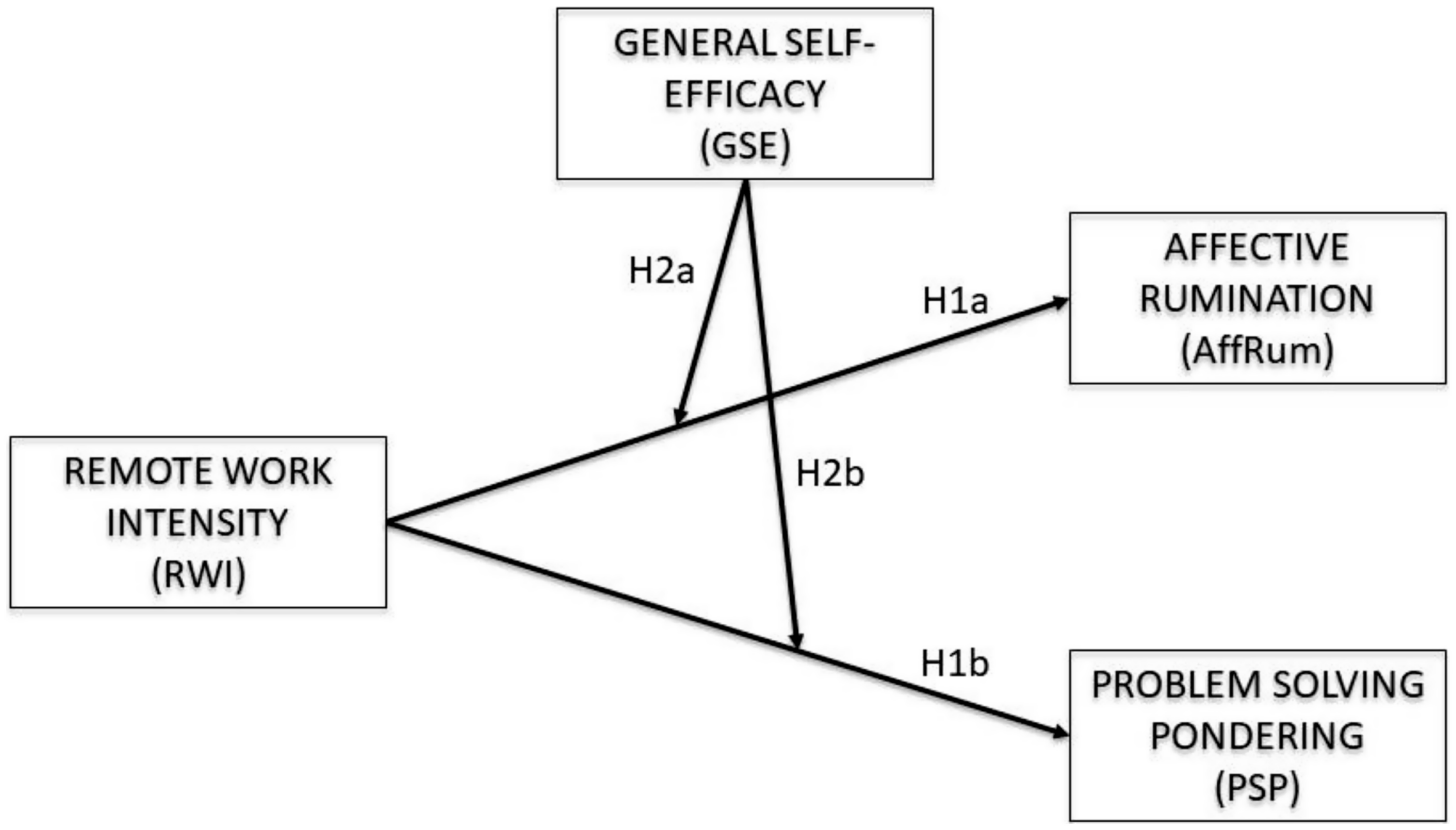
Visual representation of the study hypotheses.

Importantly, however, prior studies have predominantly examined recovery self-efficacy, defined as confidence specifically in one’s ability to detach after work (Sonnentag and Kruel, 2006), or boundary management self-efficacy as predictors of detachment outcomes. The present study takes a different theoretical position. We use general self-efficacy (GSE) (Schwarzer, 1995) as a moderator of a structural work condition (RWI) rather than as a direct predictor, consistent with the view of GSE as a broad and context-general personal resource (Chen et al., 2001). This choice is theoretically grounded in three steps. First, GSE reflects a foundational personal resource (Hobfoll, 1989) that underpins broad coping capacity across diverse and novel situations, precisely the type of multi-situational demand that characterises varied levels of hybrid work. Recovery self-efficacy is narrower, domain-specific, and may not capture the full range of adaptive regulation challenges associated with intensive remote work (i.e., managing digital boundaries, autonomous scheduling, and reduced social scaffolding). Second, the RWI context amplifies demands for autonomous self-regulation in ways that go beyond simply disconnecting after work (Mazmanian et al., 2013); they include managing ICT expectations, structuring workday boundaries without institutional cues, and sustaining performance without co-presence. Within the JD-R framework, self-efficacy is commonly conceptualized as a personal resource that helps employees manage job demands and maintain functioning (Xanthopoulou et al., 2007). A further consideration concerns conceptual specificity. Recovery self-efficacy is defined, and typically measured, as confidence in one’s own ability to switch off from work (Sonnentag and Kruel, 2006), a construct that aligns more closely with psychological detachment and recovery-related cognitions than with broader trait-like resources such as GSE. Since AffRum and PSP measure ongoing work-related thoughts during off-job time, using recovery self-efficacy as a moderator could overlap conceptually. Using recovery self-efficacy as a moderator therefore risks conceptual redundancy, as a measure of perceived ability to detach would be expected, almost by definition, to relate to detachment-related cognitions regardless of RWI. Taken together, these considerations position GSE as a boundary condition that may help clarify when RWI functions as a resource rather than a demand for recovery. In this context, general self-efficacy (Schwarzer et al., 1995) acts as a broad personal resource for self-regulation across work and non-work domains. By supporting employees’ perceived capacity to manage challenges, structure boundaries, and regulate persistent work-related thoughts beyond working hours, GSE may help explain when intensive remote work is more likely to operate as a resource or as a demand for post-work cognitive recovery (Figure 1).

Therefore, we hypothesize the following:

*H2*: The positive association between remote work intensity and affective rumination (H2a) and problem-solving pondering (H2b) will be moderated by general self-efficacy, such that higher GSE weakens the extent to which remote work intensity is associated with post-work cognitive activation.

## Methods

### Procedure and participants

Data were collected via an anonymous online Qualtrics survey (10–15 min) from 747 employees across four Italian organizations (three private; automotive, consulting, payroll; and one public municipality). Participants (45.1% male, 54.1% female; M_age = 43.7, SD = 10.7) held diverse roles, with 22.2% in leadership positions and 77.2% as followers. Average organizational tenure was 16.1 years (SD = 10.7). Regarding education, 62.4% held higher or post-graduate degrees, while 37.2% completed primary or secondary education. All participants regularly worked remotely: in the previous 15 days, 33.6% worked remotely ≤3 days, 43.1% for 4–6 days, and 23.3% for ≥ 7 days, consistent with current Italian hybrid work trends (Assolombarda, 2024; Confindustria, 2024; Osservatorio Smart Working, 2024). Full demographic details are available in Supplementary Table 1.

### Measures

In addition to the sociodemographic information presented in Supplementary Table 1, measures for general self-efficacy, affective rumination, and problem-solving pondering were also administered.

All variables were measured on a 5-point Likert scale. General self-efficacy (Schwarzer, 1995) (GSE; 10 items; *α* ≥ 0.92) assessed coping abilities (i.e., ‘*Thanks to my resourcefulness, I know how to handle unforeseen situations’*). Affective rumination (AffRum; α ≥ 0.87) and problem-solving pondering (PSP; α ≥ 0.88) (Cropley et al., 2012) were measured using the WRRQ (Cropley et al., 2012; 5 items each). Sample items include ‘*I became tense when I thought about work-related issues during my free time*’ (AffRum) and *I found solutions to work-related problems in my free time*’ (PSP).

All items were presented in Italian, following a translation and back-translation procedure to ensure linguistic and conceptual equivalence.

### Analysis

We computed validity and reliability indexes of the scales for GSE, AffRum, and PSP using the Cronbach’s alphas and Omega coefficients. To further assess the distinctiveness of the study constructs, we conducted a confirmatory factor analysis (CFA) testing the hypothesized three-factor model. The results indicated a satisfactory model fit (χ^2^(162) = 1002.136; CFI = 0.964; TLI = 0.957; RMSEA = 0.084; SRMR = 0.080), supporting the adequacy of the measurement model. All indicators loaded significantly on their respective latent factors (*p* < 0.001), with standardized loadings generally in the moderate-to-high range, providing evidence of convergent validity. Moreover, correlations among latent variables were moderate (approximately ranging between −0.20 and 0.33), indicating that the constructs are related but empirically distinct, thus supporting discriminant validity.

Additionally, descriptive analyses and Pearson’s correlations were conducted (descriptive statistics, correlations, and validity and reliability indexes of the scales are reported in the descriptive statistics paragraph). To test Hypothesis H1, we ran two one-way ANOVAs using the GLM procedure in IBM SPSS Statistics (v29). RWI, measured as an ordinal variable with three levels, was treated as a categorical factor. AffRum (H1a) and PSP (H1b) were analyzed in separate models.

The three RWI categories (≤3, 4–6, and ≥7 days in the previous 15 days) were operationalised based on the empirical distribution of remote work practices documented for Italian hybrid workers in 2023–2024 (Assolombarda, 2024; Confindustria, 2024; Osservatorio Smart Working, 2024), corresponding to low- (up to approximately 1 day per week), medium- (~2 to 3 days per week), and high-intensity (majority-remote) hybrid work, respectively. This approach of deriving intensity categories from context-specific empirical distributions mirrors a strategy already applied in prior telework research, which similarly distinguished low-, moderate-, and high-intensity telework on the basis of prevailing patterns within its own national context, as previous research (Nagata et al., 2021). Furthermore, from a research design perspective, the ordinal scale neutralizes metric biases caused by differing corporate policies and allows for the effective organization and comparison of employee subgroups across different organizations. A robustness check confirming that these results hold when RWI is modeled as a continuous variable is reported in Supplementary Table 4.

To address the potential influence of organizational-level clustering and individual-level demographic factors, organization, sex, and tenure were included as covariates in the PROCESS moderation models (*N* = 703). Full model statistics are reported in the Supplementary Tables 2, 3.

Finally, we ran the PROCESS macro (model 1) to test the moderation hypotheses (H2a and H2b) through Ordinary Least Squares (OLS) regression. Additionally, we mean-centered the GSE, AffRum, and PSP to make the interpretation of the results easier. Because RWI was a multicategorical variable with three levels, indicator (dummy) coding was applied, with employees working remotely ≤3 days in the previous 15 days serving as the reference group. This group was treated as the baseline category (coded as 0), so that the regression coefficients for the other two RWI categories (i.e., medium and high-intensity) categories represented differences in the outcome variables relative to this low-intensity remote work condition.

The ‘mcx = 1’ option was used to instruct PROCESS that the independent variable was a multi-categorical variable and dummy coding was used. Then we ran PROCESS Model 1 with 5,000 bootstrap samples and 95% confidence intervals. Model 1 was performed twice, one for each dependent variable.

## Results

### Descriptive statistics, reliability, and correlations among study variables

All multi-item scales showed excellent internal consistency, with McDonald’s Omega coefficients (*ω*) ranging from 0.88 to 0.92 (AffRum ω = 0.88; PSP ω = 0.88; GSE ω = 0.92). GSE was significantly and negatively correlated with AffRum (r = −0.16, *p* < 0.01) and positively correlated with PSP (*r* = 0.10, *p* < 0.01); these two constructs were also positively correlated with each other (*r* = 0.33, *p* < 0.01). In contrast, RWI was not significantly correlated with any of the other study variables.

The descriptive findings suggest that higher levels of GSE are related to employees being more likely to think about work-related problems outside of working hours (PSP; M = 2.93, SD = 0.91), and vice versa. Furthermore, GSE exhibits a negative correlation with AffRum (M = 3.05, SD = 0.90). This suggests that employees who perceive themselves as more self-efficacious (GSE; M = 3.91, SD = 0.58) are less likely to dwell on work-related events that elicit negative emotions outside of work, and vice versa.

In line with the literature on recovery behaviours, and as supported by the CFA, AffRum and PSP result in conceptually distinct constructs, although moderately correlated (r = 0.33). This seems to confirm that both represent individual processes that typically characterise the initial cognitive stage of post-work adjustment, yet they differ in nature and potentially in their relation to individuals’ resources, such as psychological self-efficacy.

### Hypothesis testing

A series of one-way analyses of variance (ANOVAs) were conducted to test H1a and H1b. For AffRum (H1a),mean levels were highly similar across the three remote work intensity groups: ≤3 days (M = 3.00, SD = 0.77), 4–6 days (M = 3.07, SD = 0.92), and ≥ 7 days (M = 3.08, SD = 1.03). The omnibus test was not significant, *F*(2, 705) = 0.52, *p* = 0.595, η^2^ = 0.001. Consistent with this result, Bonferroni-adjusted pairwise comparisons indicated no significant differences between any of the groups (*p* > 0.05).

A comparable pattern emerged for problem-solving pondering (H1b). Group means again showed minimal variation with values of M = 2.97 (SD = 0.89) for the ≤3 days group, M = 2.89 (SD = 0.91) for the 4–6 days group, and M = 2.97 (SD = 0.95) for the ≥ 7 days group. The ANOVA was non-significant, F(2, 705) = 0.78, *p* = 0.460, η^2^ = 0.002. Likewise, none of the Bonferroni comparisons were significant (*p* ≥ 0.77). Overall, these findings provide no evidence in support of Hypotheses H1a or H1b.

Regarding the testing of the moderation model between RWI and AffRum (H2a), the overall model (controlling for organization, sex, and tenure; N = 703) was significant, R = 0.27, R^2^ = 0.07; *F* (8, 694) = 6.77, *p* < 0.001. The interaction between RWI and GSE was significant, ΔR^2^ = 0.03, *F* (2, 695) = 12.98, p < 0.001, indicating that the effect of RWI on AffRum differed across levels of GSE.

The moderation analysis, tested for answering H2a, indicated that the association between RWI operationalized as the number of days worked remotely in the previous 15 days, and AffRum varied as a function of GSE. As discussed, RWI was modeled as a three-level categorical variable, with the reference group comprising employees who worked remotely ≤3 days in the previous 15 days; the other groups included employees who worked remotely 4–6 days and those who worked remotely 7 days or more.

Conditional effects were examined at low (−1 SD; GSE = −0.51), mean (GSE = −0.01), and high (+1 SD; GSE = 0.59) levels of GSE and are reported as differences in affective rumination relative to the reference group.

**Figure 2:**
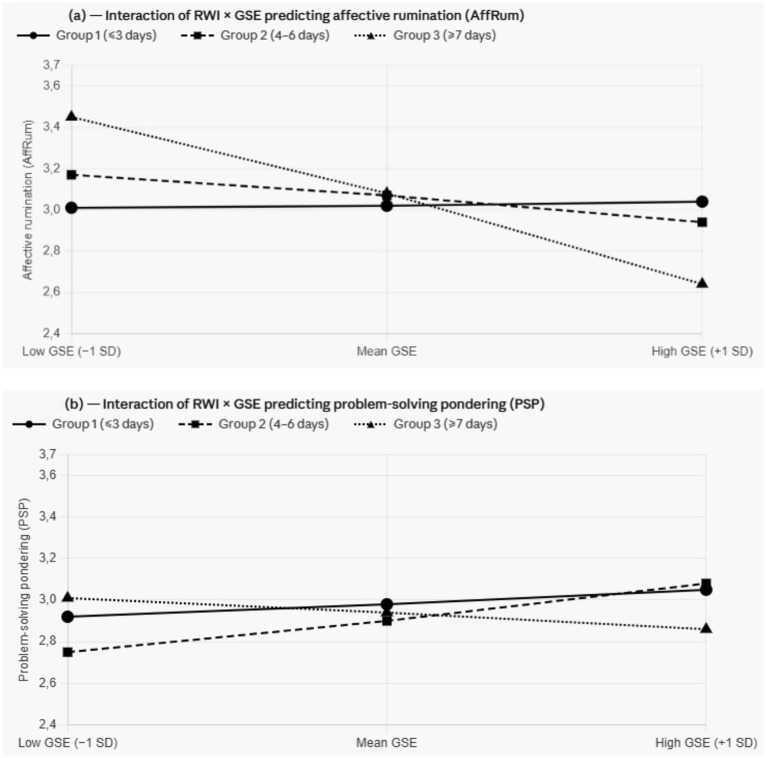
**(a)** Interaction plot of remote work intensity (RWI) and general self-efficacy (GSE) predicting affective rumination (AffRum). Lines represent predicted AffRum values for the three RWI groups (Group 1: ≤3 days; Group 2: 4–6 days; Group 3: ≥7 days worked remotely in the previous 15 days) at low (−1 SD, GSE = −0.51), mean (GSE = −0.01), and high (+1 SD, GSE = 0.59) levels of general self-efficacy. Values are estimated means from the moderation model controlling for organization, sex, and tenure (*N* = 703). **(b)** Interaction plot of remote work intensity (RWI) and general self-efficacy (GSE) predicting problem-solving pondering (PSP). Note. Lines represent predicted PSP values for the three RWI groups (Group 1: ≤3 days; Group 2: 4–6 days; Group 3: ≥7 days worked remotely in the previous 15 days) at low (−1 SD, GSE = −0.51), mean (GSE = −0.01), and high (+1 SD, GSE = 0.59) levels of general self-efficacy. Values are estimated means from the moderation model controlling for organization, sex, and tenure (*N* = 703). No significant RWI × GSE interaction was observed.

At low GSE, employees who worked remotely ≥ 7 days reported significantly higher affective rumination than the reference group (b = 0.44, SE = 0.12, p < 0.001), whereas the 4–6 days group did not significantly differ from the reference group (b = 0.16, SE = 0.10, *p* = 0.107). At mean GSE, neither the 4–6 days group (b = 0.04, SE = 0.08, *p* = 0.574) nor the ≥ 7 days group (b = 0.06, SE = 0.09, *p* = 0.470) differed significantly from the reference group. At high GSE, the pattern reversed for the ≥ 7 days group, which reported significantly lower affective rumination than the reference group (b = −0.40, SE = 0.12, *p* = 0.001), while the 4–6 days group again did not differ significantly from the reference group (b = −0.10, SE = 0.11, *p* = 0.349). Full regression coefficients and model statistics are reported in Supplementary Table 2. Overall, these findings support Hypothesis 2a, indicating that the association between RWI and AffRum varies as a function of GSE. Specifically, this happens when comparing employees working remotely ≤3 days, with those working remotely ≥7 days reported higher AffRum at low GSE, no significant differences at mean GSE, and lower AffRum at high GSE (see Figure 2a).

To answer H2b we tested whether GSE moderated the association between RWI and PSP. The overall model (controlling for organization, sex, and tenure; N = 703) explained a small but significant proportion of variance in PSP (R^2^ = 0.04, R = 0.20, *F*(8, 694) = 3.46, p = 0.001). However, the interaction terms were not statistically significant (Group 2 × GSE: b = 0.18, SE = 0.14, *p* = 0.192; Group 3 × GSE: b = −0.26, SE = 0.16, *p* = 0.104), indicating no clear evidence that the effect of RWI on PSP varies as a function of GSE.

Conditional effects were probed at low (−1 SD; GSE = −0.51), mean (GSE = −0.01), and high (+1 SD; GSE = 0.59) levels of GSE and are reported as differences relative to the reference group. At low GSE, neither Group 2 (b = −0.17, SE = 0.11, *p* = 0.115) nor Group 3 (b = 0.09, SE = 0.13, *p* = 0.460) differed significantly from the reference group. At mean GSE, the contrasts were again non-significant for Group 2 (b = −0.08, SE = 0.08, *p* = 0.327) and Group 3 (b = − 0.03, SE = 0.09, *p* = 0.716). At high GSE, neither Group 2 (b = 0.03, SE = 0.11, *p* = 0.796) nor Group 3 (b = −0.19, SE = 0.13, *p* = 0.142) differed significantly from the reference group. Overall, these findings do not support a reliable moderating role of GSE in the relationship between RWI and PSP. Full regression coefficients and model statistics are reported in Supplementary Table 3.

Consistent with this result, simple slope analyses showed that the relationship between RWI and PSP was not significant at any level of the moderator across the three hybrid work groups (see Figure 2b). Taken together, these results fail to support Hypothesis 2b.

## Discussion

We investigated the link between RWI and two distinct forms of after-hours cognition: AffRum and PSP. Furthermore, we explored whether GSE acts as a boundary condition for these associations. Collectively, these variables represent the most proximal cognitive pathways linking hybrid work to recovery outcomes (Cropley et al., 2012). The findings show a clear divergence between the two outcomes. For AffRum, RWI mattered only in combination with GSE: compared with employees working remotely ≤ 3 days, those working remotely ≥ 7 days reported higher AffRum when GSE was low, no differences at mean GSE, and lower AffRum when GSE was high. This crossover pattern suggests that the number of remote workdays is not inherently good or bad for recovery; rather, it becomes a risk condition for employees with fewer personal resources.

The non-significant findings for H1 are consistent with this picture. The absence of a direct main effect of RWI on either AffRum or PSP is not theoretically surprising in light of the interaction pattern. The null findings for H1 thus reinforce rather than undermine the central argument, RWI alone is an insufficient predictor of recovery-related cognitions, and its consequences become interpretable only when personal resources are accounted for.

These results can be interpreted through the lens of the autonomy paradox (Kossek et al., 2006; Delanoeije and Verbruggen, 2020; Miglioretti et al., 2021; Donati et al., 2025; Zhou, 2020). Autonomy is here indirectly reflected in a higher number of remote working days, and it helps fulfill our need for autonomy (Deci and Ryan, 2014). RWI, in fact, as discussed, can be understood as a structural work arrangement that may increase employees’ exposure to autonomy-related working conditions (Contreras et al., 2020; Donati et al., 2025). Increased autonomy may also increase difficulties in maintaining boundaries and, in turn, foster AffRum. Importantly, however, this pattern emerged primarily among employees with low GSE. As GSE increased to average and high levels, AffRum decreased, highlighting GSE as a resource-buffering factor (Sonnentag and Kruel, 2006; Sonnentag and Fritz, 2007; Cropley and Zijlstra, 2011; Demerouti et al., 2012; Cropley et al., 2023; Waddington, 2023) under conditions of intensive remote work. It is important to note, however, that this cross-sectional pattern should not be interpreted as evidence of causal protection: among employees with high GSE, intensive remote work was comparatively associated with lower AffRum relative to the low-intensity reference group, a pattern consistent with a buffering interpretation but not with conclusions about dynamic recovery processes unfolding over time.

The cross-sectional design precludes any causal inference: it is equally plausible that employees who were already experiencing lower AffRum self-selected into high-intensity remote work arrangements, or that unmeasured third variables (e.g., job autonomy, workload, or organizational culture) account for the observed pattern. High GSE shows that the cross-sectional association between high RWI and AffRum differs as a function of GSE level, with employees at the lower end of the GSE distribution showing a more unfavourable pattern.

Consistent with Conservation of Resources (COR) theory (Hobfoll, 1989), intensive remote work may transition from a resource to a demand that blurs work-life boundaries, as evidenced by increased AffRum. These results highlight the importance of personal resources, with GSE serving as a buffer for effective resource management. This suggests a necessary shift in how we perceive RWI: remote work frequency is not a negative predictor per se. Although challenges such as blurred boundaries remain, remote work can function as a positive resource when it provides flexibility without compromising well-being (Miglioretti et al., 2021; Margheritti et al., 2023). Accordingly, when higher RWI appears to coincide with greater AffRum, future work should examine the quality and type of remote work arrangements in place (Nagata et al., 2021; Soga et al., 2022). From a recovery perspective (Cropley et al., 2023), it is essential that hybrid work is designed and enacted in a more agile, high-quality manner (Miglioretti, 2023).

The crossover pattern observed for AffRum warrants deeper theoretical consideration, particularly the finding that high-RWI employees with high GSE reported lower AffRum than the low-RWI reference group. This pattern can be understood through the lens of cognitive appraisal theory (Lazarus and Folkman, 1984). According to this framework, the stress experienced in response to a demanding situation depends not only on primary appraisal (whether the situation is perceived as threatening) but critically on secondary appraisal, that is, the individual’s evaluation of their coping resources relative to the demands at hand. Self-efficacy is a central component of secondary appraisal (Bandura, 1994; Schwarzer and Hallum, 2008): individuals with high GSE assess themselves as possessing sufficient internal resources to manage situational demands, which reduces the likelihood that those demands will be experienced as threatening or uncontrollable. In the context of intensive remote work, this means that employees with high GSE may appraise the autonomy-related demands, connectivity requirements, and boundary management challenges associated with high RWI not as threatening but as manageable, or even as affording greater discretion over their work schedules. This challenge appraisal, rather than threat appraisal, may attenuate the emotional activation underlying affective rumination, thereby suppressing the carry-over of work-related distress into off-job time (Lazarus and Folkman, 1984; Demerouti et al., 2012). Consistent with this interpretation, evidence from self-efficacy research shows that highly efficacious individuals focus attention on task demands and mobilise effective coping strategies under pressure, rather than dwelling on potential failure or negative outcomes (Bandura, 1994). In a hybrid work context, this translates into more effective boundary management (Mazmanian et al., 2013). The comparatively lower AffRum at high GSE and high RWI may therefore reflect not that intensive remote work is inherently beneficial, but that employees with strong general coping resources are better positioned to leverage its autonomy-related advantages while managing its demands.

Finally, it is noteworthy that GSE did not function as a comparable buffering factor for PSP. Two complementary explanations may account for this pattern. First, PSP is primarily goal-directed cognition (Kinnunen et al., 2017). It is mobilised by task incompletion or performance goals rather than by the structural variable of how many days one works remotely. This may explain why varying the dose of RWI does not differentially activate PSP across GSE levels. However, it remains crucial to consider that over time, even problem-solving pondering (PSP) can give rise to persistent work-related rumination, with subsequent negative triggers for employee well-being (Querstret and Cropley, 2012). Second, the WRRQ’s PSP subscale captures a range of solution-focused cognitions, some of which may be appraised as functional (and therefore not systematically suppressed by higher self-efficacy) while others may shade into perseverative thinking when problems remain unresolved (Syrek et al., 2017). This internal heterogeneity within the PSP construct could dilute the moderating signal of GSE, yielding null interactions even if some PSP episodes are in fact moderated. These explanations are consistent with the literature characterising PSP as the less problematic component within the rumination framework (Cropley et al., 2023).

This suggests that models of remote work and recovery should explicitly integrate personal resources as boundary conditions, rather than assuming uniform effects of remote work intensity.

### Limitation and future research

While providing valuable insights, this study’s cross-sectional design limits our ability to establish definitive causal relationships. Although the model is grounded in theory, reciprocal effects cannot be ruled out. To better capture these temporal dynamics, future research should employ longitudinal or diary designs, allowing for a more granular examination of how rumination and recovery fluctuate daily. Because all psychological variables were collected from the same source at a single time point, common method bias represents a potential limitation. Several features of the study design mitigate the most severe forms of common method bias: the predictor variable (RWI) is a behavioural frequency count less susceptible to socially desirable responding than attitude or affect items; the constructs differ in content, valence, and temporal orientation, reducing the risk of uniform response-tendency inflation; and the obtained correlations are modest and theoretically patterned. Nevertheless, future research should employ multi-source designs (i.e., significant others’ ratings of after-work recovery behaviour), longitudinal panel studies, or ecological momentary assessment approaches to overcome this limitation. Such designs would also enable examination of temporal dynamics in the RWI-rumination relationship, which the present study cannot address.

Second, although the sample size is adequate, data collection was limited to three private firms and one public administration. This composition suggests that sectoral norms may have influenced the findings. Future research should include a broader range of sectors to enhance generalizability and enable more robust comparisons. Sensitivity analyses including organization, tenure, and sex as covariates confirmed that the core RWI × GSE interaction patterns remained fully stable and statistically significant for both outcomes, demonstrating that these psychological mechanisms hold regardless of the specific company context. While the direct effect of the organization variable hovered near marginal significance (with *p*-values of 0.064 for AffRum and 0.055 for PSP), its inclusion did not confound our main findings. However, since our sample was drawn from only four organizations, which precludes formal multilevel modelling to explicitly partition within- and between-firm variance, future research should replicate these models using large-scale, multi-organization samples. Furthermore, the modest effect size (total R^2^ = 0.07; interaction ΔR^2^ = 0.03) indicates that many individual, job-level, and contextual factors beyond those examined here may shape post-work rumination. Although a ΔR^2^ = 0.03 for the interaction term is common and theoretically meaningful in field studies testing moderation effects, it highlights that future research should include further predictors and moderators (i.e., workload and social support) to capture a wider proportion of variance.

Regarding the measures, while this study provides a rationale for using general self-efficacy (GSE) over recovery self-efficacy, future research should compare these constructs directly within the same model. Such a comparison would clarify if broad resources like GSE and domain-specific recovery resources explain different aspects of detachment-related thoughts or if the current results stem from general self-regulatory capacity or a belief in disconnecting from work.

Finally, a limitation of the present study lies in having sacrificed the quantitative granularity of a continuous variable in favor of an ordinal scale. However, this choice was discussed rationally and it was necessary to standardize comparisons across heterogeneous organizational contexts and subgroups with differing access rights to remote work, thereby capturing the specific organizational baseline. Future research conducted on fully representative national samples should test remote work intensity as a continuous variable, exploring whether hourly or daily precision might reveal further nuances within the categories that are by now well-established in institutional practice (i.e., International Labour Organization (ILO), 2021; Assolombarda, 2024; Confindustria, 2024).

### Practical and theoretical implication

These findings suggest that although remote work intensity plays a key role in the recovery process, it must be analyzed alongside individual differences in personal resources.

The interaction pattern observed for affective rumination indicates that the cognitive costs of intensive remote work may be unevenly distributed across employees, being more pronounced among those with lower general self-efficacy. While the cross-sectional design precludes causal conclusions, this pattern is consistent with the idea that recovery-oriented hybrid work management should attend to employee resource levels, not only to the frequency of remote days.

At the individual level, these findings point to the potential value of interventions targeting self-regulatory resources. Programs aimed at strengthening employees’ general coping capacity, for example, through training in boundary management or cognitive detachment strategies, may be particularly relevant for employees working at higher remote intensities (Mazmanian et al., 2013), especially with fewer personal resources. Such initiatives warrant empirical evaluation in longitudinal or intervention designs before firm recommendations can be made.

At the organizational level, the findings are consistent with the broader argument that recovery support in hybrid settings should not rely solely on individual self-regulation (Mazmanian et al., 2013). Organisational dynamics, including team norms around availability and responsiveness, are likely to shape recovery outcomes independently of individual resources (Parker et al., 2026). Right-to-disconnect policies serve as effective boundary resources only when translated into concrete routines (Barber and Santuzzi, 2015). These measures may be particularly relevant when intensive remote work becomes riskier for employees with fewer personal resources, as suggested by the interaction pattern observed for affective rumination. For example, cultures that implicitly reward availability or encourage rapid responsiveness may intensify rumination and reduce opportunities for restorative activities (Parker et al., 2026; Cropley et al., 2023). Taken together, these observations suggest that hybrid work governance should move beyond time-based metrics toward designs that actively support psychological disengagement, though replication in longitudinal and multi-organization samples is needed before specific managerial recommendations can be advanced with confidence.

## Conclusion

The present study reveals that the complexity of hybrid work management lies in its impact on recovery processes. Rather than focusing solely on the number of remote days, our results highlight the role of general self-efficacy as a key moderator that shapes the link between remote work intensity and recovery-related cognitions. Ultimately, remote work should not be viewed as universally positive or negative; its implications are largely defined by the interplay between job demands and the individual’s resource pool.

## Data Availability

The datasets used and analysed during the current study are available from the corresponding author on reasonable request.

## References

[ref1] Allen T. D. Golden T. D. Shockley K. M. (2015). How effective is telecommuting? Assessing the status of our scientific findings. Psychol. Sci. Public Interest 16, 40–68. doi: 10.1177/1529100615593273, 26403188

[ref2] Assolombarda (2024). Assolombarda - Smart working in numeri - 2024 Assolombarda.it. Available online at: https://www.assolombarda.it/centro-studi/smart-working-in-numeri-2024 (Accessed February 19, 2026).

[ref3] Baas M. De Dreu C. K. W. Nijstad B. A. (2008). A meta-analysis of 25 years of mood-creativity research: hedonic tone, activation, or regulatory focus? Psychol. Bull. 134, 779–806. doi: 10.1037/a0012815, 18954157

[ref4] Bandura A. (1994). “Self-efficacy,” in Encyclopedia of Human Behavior, ed. Ramachaudran V. S., vol. 4 (New York: Academic Press), 71–81. Reprinted in H. Friedman [Ed.], Encyclopedia of mental health. San Diego: Academic Press, 1998

[ref5] Barber L. K. Santuzzi A. M. (2015). Please respond ASAP: workplace telepressure and employee recovery. J. Occup. Health Psychol. 20, 172–189. doi: 10.1037/a0035043, 25365629

[ref6] Belle S. M. Burley D. L. Long S. D. (2015). Where do I belong? High-intensity teleworkers’ experience of organizational belonging. Hum. Resour. Dev. Int. 18, 76–96. doi: 10.1080/13678868.2014.979006

[ref7] Bloom N. Han R. Liang J. (2024). Hybrid working from home improves retention without damaging performance. Nature 630, 920–925. doi: 10.1038/s41586-024-07500-2, 38867040 PMC11208135

[ref8] Brosschot J. F. Gerin W. Thayer J. F. (2006). The perseverative cognition hypothesis: a review of worry, prolonged stress-related physiological activation, and health. J. Psychosom. Res. 60, 113–124. doi: 10.1016/j.jpsychores.2005.06.074, 16439263

[ref9] Chen G. Gully S. M. Eden D. (2001). Validation of a new general self-efficacy scale. Organ. Res. Methods. 4, 62–83. doi: 10.1177/109442810141004

[ref10] Confindustria (2024) Indagine Confindustria sul lavoro del 2024 | Confindustria. Available online at: https://www.confindustria.it/pubblicazioni/indagine-confindustria-sul-lavoro-del-2024/ (Accessed February 19, 2026).

[ref11] Contreras F. Baykal E. Abid G. (2020). E-leadership and teleworking in times of COVID-19 and beyond: what we know and where do we go. Front. Psychol. 11:590271. doi: 10.3389/fpsyg.2020.590271, 33362656 PMC7759482

[ref12] Cropley M. Michalianou G. Pravettoni G. Millward L. J. (2012). The relation of post-work ruminative thinking with eating behaviour. Stress. Health 28, 23–30. doi: 10.1002/smi.1397, 22259155

[ref13] Cropley M. Rydstedt L. W. Chelidoni O. Ollis L. Querstret D. (2023). Work-related rumination declines with age but is moderated by gender. Work 76, 587–594. doi: 10.3233/WOR-220288, 36872828

[ref14] Cropley M. Zijlstra F. R. H. (2011). “Work and rumination,” in Handbook of Stress in the Occupations, eds. Langan-Fox J. Cooper C. (Cheltenham: Edward Elgar Publishing).

[ref15] Deci E. L. Ryan R. M. (2014). “Autonomy and need satisfaction in close relationships: relationships motivation theory,” in Human Motivation and Interpersonal Relationships, ed. Weinstein N. (Dordrecht: Springer Netherlands), 53–73.

[ref16] Delanoeije J. Verbruggen M. (2020). Between-person and within-person effects of telework: a quasi-field experiment. Eur. J. Work Organ. Psychol. 29, 795–808. doi: 10.1080/1359432X.2020.1774557

[ref17] Demerouti E. Bakker A. B. Sonnentag S. Fullagar C. J. (2012). Work-related flow and energy at work and at home: a study on the role of daily recovery. J. Organ. Behav. 33, 276–295. doi: 10.1002/job.760

[ref18] Demerouti E. Derks D. Ten Brummelhuis L. L. Bakker A. B. (2014). “New ways of working: impact on working conditions, work–family balance, and well-being,” in The Impact of ICT on Quality of Working Life, eds. Korunka C. Hoonakker P. (Dordrecht: Springer Netherlands), 123–141.

[ref19] Derks D. Van Duin D. Tims M. Bakker A. B. (2015). Smartphone use and work–home interference: the moderating role of social norms and employee work engagement. J. Occup. Organ. Psychol. 88, 155–177. doi: 10.1111/joop.12083

[ref20] Donati S. Toscano F. Zappalà S. (2025). Advantage of remote workstation and job performance: the impact of worktime autonomy and remote work intensity. Ergonomics 68, 1829–1843. doi: 10.1080/00140139.2024.2439914, 39686851

[ref21] Eden D. (2001). Vacations and other respites: studying stress on and off the job, in Well-Being in Organizations. eds. C. Cooper and I. T. Robertson (West Sussex, UK: John Wiley & Sons, Ltd), 305–330.

[ref22] Etzion D. Eden D. Lapidot Y. (1998) Relief from Job Stressors and Burnout: Reserve Service as a Respite. Washington, DC: American Psychological Association. 10.1037/0021-9010.83.4.577 9729927

[ref23] Gajendran R. S. Ponnapalli A. R. Wang C. Javalagi A. A. (2024). A dual pathway model of remote work intensity: a meta-analysis of its simultaneous positive and negative effects. Pers. Psychol. 77, 1351–1386. doi: 10.1111/peps.12641

[ref24] Gratton L. (2021) Four Principles to Ensure Hybrid Work Is Productive Work – ProQuest. Available online at: https://www.proquest.com/openview/3db08d55624c73da70bf8208bc4a9081/1?pq-origsite=gscholar&cbl=26142 (Accessed January 29, 2026).

[ref25] Heiden M. Widar L. Wiitavaara B. Boman E. (2021). Telework in academia: associations with health and well-being among staff. High. Educ. 81, 707–722. doi: 10.1007/s10734-020-00569-4

[ref26] Hislop D. Axtell C. (2009). To infinity and beyond?: workspace and the multi-location worker. New Technol. Work Employ. 24, 60–75. doi: 10.1111/j.1468-005X.2008.00218.x

[ref27] Hobfoll S. E. (1989) Conservation of resources: A new attempt at conceptualizing stress. American Psychologist, 44, 513–524. doi: 10.1037/0003-066X.44.3.513 2648906

[ref28] Hobfoll S. E. (2011). Conservation of resources theory: Its implication for stress, health, and resilience. In S. Folkman (Ed.), The Oxford Handbook of Stress, Health, and Coping, (New York, NY: Oxford University Press), 127–147.

[ref29] International Labour Organization (ILO). (2021). Working from home: From invisibility to decent work. Geneva: International Labour Office. Available online at: https://researchrepository.ilo.org/esploro/outputs/report/Working-from-home-from-invisibility-to/995218635902676

[ref30] Junker N. M. Baumeister R. F. Straub K. Greenhaus J. H. (2021). When forgetting what happened at work matters: the role of affective rumination, problem-solving pondering, and self-control in work–family conflict and enrichment. J. Appl. Psychol. 106, 1750–1766. doi: 10.1037/apl0000847, 33090861

[ref31] Kinnunen U. Feldt T. Sianoja M. De Bloom J. Korpela K. Geurts S. (2017). Identifying long-term patterns of work-related rumination: associations with job demands and well-being outcomes. Eur. J. Work Organ. Psychol. 26, 514–526. doi: 10.1080/1359432X.2017.1314265

[ref32] Kniffin K. M. Narayanan J. Anseel F. Antonakis J. Ashford S. P. Bakker A. B. . (2021). COVID-19 and the workplace: implications, issues, and insights for future research and action. Am. Psychol. 76, 63–77. doi: 10.1037/amp0000716, 32772537

[ref33] Kossek E. E. Lautsch B. A. Eaton S. C. (2006). Telecommuting, control, and boundary management: correlates of policy use and practice, job control, and work–family effectiveness. J. Vocat. Behav. 68, 347–367. doi: 10.1016/j.jvb.2005.07.002

[ref34] Lazarus R. S. Folkman S. (1984). Stress, Appraisal, and Coping. New York, NY: Springer.

[ref35] Margheritti S. Negrini A. Miglioretti M. (2023). Can psychological capital promote safety behaviours? A systematic review. Int. J. Occup. Saf. Ergon. 29, 1451–1459. doi: 10.1080/10803548.2022.2135285, 36221859

[ref36] Mazmanian M. Orlikowski W. J. Yates J. (2013). The autonomy paradox: the implications of Mobile email devices for knowledge professionals. Organ. Sci. 24, 1337–1357. doi: 10.1287/orsc.1120.0806, 19642375

[ref37] McEwen B. S. (1998). Protective and damaging effects of stress mediators. N. Engl. J. Med. 338, 171–179. doi: 10.1056/NEJM199801153380307, 9428819

[ref38] Miglioretti M. (2023). Telework quality and employee well-being: lessons learned from the COVID-19 pandemic in Italy - Miglioretti - 2023 - New Technology, Work and Employment - Wiley Online Library. doi: 10.1111/ntwe.12263 PMC9877874 36718468

[ref39] Miglioretti M. Gragnano A. Margheritti S. Picco E. (2021). Not all telework is valuable. Rev. Psicol. Trab. Organ. 37, 11–19. doi: 10.5093/jwop2021a6

[ref40] Nagata T. Nagata M. Ikegami K. Hino A. Tateishi S. Tsuji M. . (2021). Intensity of home-based telework and work engagement during the COVID-19 pandemic. J. Occup. Environ. Med. 63, 907–912. doi: 10.1097/JOM.0000000000002299, 34334780 PMC8562918

[ref41] Osservatorio Smart Working (2024) Lo Smart Working in Italia Oss. Digit. Innov. Politec. Milano. Available online at: https://www.osservatori.net/report/smart-working/lo-smart-working-in-italia/ (Accessed February 19, 2026).

[ref42] Parker S. L. Dawson N. Keenan C. Sonnentag S. Neal A. Jimmieson N. L. (2026). Hybrid work and daily energy dynamics: breaks, inclusion, and evening recovery. Eur. J. Work Organ. Psychol. 20, 1–20. doi: 10.1080/1359432X.2026.2622933, 37339054

[ref43] Querstret D. Cropley M. (2012). Exploring the relationship between work-related rumination, sleep quality, and work-related fatigue. J. Occup. Health Psychol. 17, 341–353. doi: 10.1037/a0028552, 22746369

[ref44] Rodríguez-Modroño P. (2022). Working conditions and work engagement by gender and digital work intensity. Information 13:277. doi: 10.3390/info13060277

[ref45] Rumsey M. G. Walker C. B. Harris J. H. (2013). Personnel Selection and Classification. Hillsdale, New Jersey: Psychology Press.

[ref46] Schwarzer (1995). The General Self. Available online at: https://userpage.fu-berlin.de/health/engscal.htm (Accessed February 19, 2026).

[ref47] Schwarzer R. Hallum S. (2008). Perceived teacher self-efficacy as a predictor of job stress and burnout: mediation analyses. Appl. Psychol. 57, 152–171. doi: 10.1111/j.1464-0597.2008.00359.x

[ref48] Soga L. R. Bolade-Ogunfodun Y. Mariani M. Nasr R. Laker B. (2022). Unmasking the other face of flexible working practices: a systematic literature review. J. Bus. Res. 142, 648–662. doi: 10.1016/j.jbusres.2022.01.024

[ref49] Sonnentag S. (2018). The recovery paradox: portraying the complex interplay between job stressors, lack of recovery, and poor well-being. Res. Organ. Behav. 38, 169–185. doi: 10.1016/j.riob.2018.11.002, 38826717

[ref50] Sonnentag S. Fritz C. (2007). The recovery experience questionnaire: development and validation of a measure for assessing recuperation and unwinding from work. J. Occup. Health Psychol. 12, 204–221. doi: 10.1037/1076-8998.12.3.204, 17638488

[ref51] Sonnentag S. Fritz C. (2015). Recovery from job stress: the stressor-detachment model as an integrative framework: the stressor-detachment model. J. Organ. Behav. 36, S72–S103. doi: 10.1002/job.1924

[ref52] Sonnentag S. Kruel U. (2006). Psychological detachment from work during off-job time: the role of job stressors, job involvement, and recovery-related self-efficacy. Eur. J. Work Organ. Psychol. 15, 197–217. doi: 10.1080/13594320500513939

[ref53] Spoor E. de Jonge J. Hamers J. P. (2010). Design of the DIRECT-project: interventions to increase job resources and recovery opportunities to improve job-related health, well-being, and performance outcomes in nursing homes. BMC Public Health 10:293. doi: 10.1186/1471-2458-10-293, 20509923 PMC2893094

[ref54] Spreitzer G. M. Cameron L. Garrett L. (2017). Alternative work arrangements: two images of the new world of work. Annu. Rev. Organ. Psychol. Organ. Behav. 4, 473–499. doi: 10.1146/annurev-orgpsych-032516-113332

[ref55] Syrek C. J. Weigelt O. Peifer C. Antoni C. H. (2017). Zeigarnik’s sleepless nights: how unfinished tasks at the end of the week impair employee sleep on the weekend through rumination. J. Occup. Health Psychol. 22, 225–238. doi: 10.1037/ocp0000031, 27101340

[ref56] Timms C. Brough P. O’Driscoll M. Kalliath T. Siu O. L. Sit C. . (2015). Flexible work arrangements, work engagement, turnover intentions and psychological health. Asia Pac. J. Hum. Resour. 53, 83–103. doi: 10.1111/1744-7941.12030

[ref57] Vahle-Hinz T. Mauno S. De Bloom J. Kinnunen U. (2017). Rumination for innovation? Analysing the longitudinal effects of work-related rumination on creativity at work and off-job recovery. Work Stress. 31, 315–337. doi: 10.1080/02678373.2017.1303761

[ref58] Waddington J. (2023). Self-efficacy. ELT J. 77, 237–240. doi: 10.1093/elt/ccac046

[ref59] Weigelt O. Syrek C. J. Schmitt A. Urbach T. (2019). Finding peace of mind when there still is so much left undone—a diary study on how job stress, competence need satisfaction, and proactive work behavior contribute to work-related rumination during the weekend. J. Occup. Health Psychol. 24, 373–386. doi: 10.1037/ocp0000117, 29781629

[ref60] Xanthopoulou D. Bakker A. B. Demerouti E. Schaufeli W. B. (2007). The role of personal resources in the job demands-resources model. Int. J. Stress. Manag. 14, 121–141. doi: 10.1037/1072-5245.14.2.121

[ref61] Zhou E. X. (2020). The “too-much-of-a-good-thing” effect of job autonomy and its explanation mechanism. Psychology 11, 299–313. doi: 10.4236/psych.2020.112019

[ref62] Zijlstra F. R. H. Sonnentag S. (2006). After work is done: psychological perspectives on recovery from work. Eur. J. Work Organ. Psychol. 15, 129–138. doi: 10.1080/13594320500513855

