# Supplementary material for: After-work recovery in hybrid work: remote work intensity and general self-efficacy in work-related rumination
Source: Front Psychol. 2026 Jul 28;17:1845951. doi: 10.3389/fpsyg.2026.1845951 (PMC13457151; doi:10.3389/fpsyg.2026.1845951)
Supplement: Supplementary file 1 [file Data_Sheet_1.PDF]

Table 1 Socio-Demographic and Occupational Characteristics

	N
	747
Private companies	
Automotive	368
Consulting	165
Payroll and workforce	111
Public Administration	
Municipality	103
**Age** (years)	43.7±10.7 (22-65)
**Sex** (male/female/other)	337 (45.1%) / 404 (54.1%) / 6 (0.8%)
**Organizational Tenure**	16.1 ±10.7(0.8 - 43)
**Educational Level**	
Primary and Secondary Education	276(37.2%)
Higher and Post-Graduate Education	466 (62.4%)
**Role**	
Leader	166 (22.2%)
Follower	577 (77.2%)
Missing	4 (0.5%)
**Number of remote working days in the past 15 days***	
≤3 days	251 (33.6%)
4 - 6 days	322 (43.1%)
≥ 7 days	174 (23.3%)

Note: The number of remote working days was categorized into the three brackets shown in the table (≤3, 4-6, and ≥7) based on the most frequent remote work intensity observed in the Italian context (Assolombarda, 2024; Confindustria, 2024; Osservatorio Smart Working, 2024).

Table 2. Moderation results (PROCESS Model 1) predicting AffRum

**Model summary**						
**N**	R	R^2	MSE	F	Df1, df2	p
**708**	0.25	0.06	0.76	9.66	5, 702	< .001
703 *(with controls: organisation, tenure, sex)*	0.27	0.07	0.76	6.77	8, 694	< .001

**Regression coefficients**						
**Term**	b	SE	t	p	95% CI LL	95% CI UL
**Constant**	3.00	0.06	53.45	< .001	2.89	3.11
**X1 (Group 2 vs Ref)**	0.06	0.08	0.85	.398	-0.08	0.21
**X2 (Group 3 vs Ref)**	0.09	0.09	1.01	.314	-0.09	0.27
**GSE**	0.05	0.10	0.46	.647	-0.15	0.24
**X1 x GSE (int_1)**	-0.25	0.13	-1.88	.061	-0.50	0.01
**X2 x GSE (int_2)**	-0.80	0.15	-5.25	< .001	-1.09	-0.50
**Constant**	3.09	0.16	18.93	< .001	2.77	3.41
**X1 (Group 2 vs Ref)**	0.05	0.08	0.63	.527	-0.10	0.20
**X2 (Group 3 vs Ref)**	0.05	0.09	0.58	.563	-0.13	0.23
**GSE**	0.02	0.10	0.19	.849	-0.18	0.21
**X1 x GSE (int_1)**	-0.23	0.13	-1.76	.078	-0.49	0.03
**X2 x GSE (int_2)**	-0.77	0.15	-5.04	< .001	-1.07	-0.47
**Organisation (covariate)**	-0.07	0.04	-1.86	.064	-0.15	0.00
**Tenure (covariate)**	0.00	0.00	0.15	.879	-0.01	0.01
**Sex (covariate)**	0.06	0.07	0.94	.347	-0.07	0.19
**Highest-order interaction test**						
**Test**	ΔR^2	F	df1, df2	p		
**X × W (RWI x GSE)**	0.04	14.01	2, 702	< .001		
**X × W (RWI x GSE)**	0.03	12.98	2, 695	< .001		
**Conditional effects of RWI (vs reference group) at values of GSE**						
**GSE level**	GSE value	Contrast	Effect (b)	SE	p	Estimated means (Ref / G2 / G3)
**Low (≈16th pct; −1SD)**	−0.51	X1 (G2 vs Ref)	0.19	0.10	.062	2.98 / 3.17 / 3.48
		X2 (G3 vs Ref)	0.50	0.12	< .001	
**Low (≈16th pct; −1SD)**	−0.51	X1 (G2 vs Ref)	0.16	0.10	.107	3.01 / 3.17 / 3.45

		X2 (G3 vs Ref)	0.44	0.12	< .001	
**Mean (≈50th pct)**	−0.01	X1 (G2 vs Ref)	0.07	0.08	.378	3.00 / 3.07 / 3.10
		X2 (G3 vs Ref)	0.10	0.09	.269	
**Mean (≈50th pct)**	−0.01	X1 (G2 vs Ref)	0.04	0.08	.574	3.02 / 3.07 / 3.08
		X2 (G3 vs Ref)	0.06	0.09	.470	
**High (≈84th pct; +1SD)**	0.59	X1 (G2 vs Ref)	−0.08	0.11	.451	3.03 / 2.95 / 2.65
		X2 (G3 vs Ref)	−0.38	0.12	.002	
**High (≈84th pct; +1SD)**	0.59	X1 (G2 vs Ref)	−0.10	0.11	.349	3.04 / 2.94 / 2.64
		X2 (G3 vs Ref)	−0.40	0.12	.001	

Note: X was indicator-coded with the reference group coded as X1 = 0, X2 = 0 (Group 1: ≤3 remote days). Group 2: X1 = 1, X2 = 0; Group 3: X1 = 0, X2 = 1. GSE was mean-centered; conditional tables use the 16th, 50th, and 84th percentiles of GSE as probe values.

Values highlighted in blue are from the sensitivity model including organisation, tenure, and sex as covariates (N = 703). The RWI × GSE interaction remained significant in both models.

Table 3. Moderation results (PROCESS Model 1) predicting PSP

**Model summary**						
N	R	R²	MSE	F	Df1, df2	p
**708**	0.15	0.02	0.81	3.43	5, 702	.005
703 *(with controls: organisation, tenure, sex)*	0.20	0.04	0.81	3.46	8, 694	.001
**Regression coefficients**						
**Term**	b	SE	t	p	95% CI LL	95% CI UL
**Constant**	2.99	0.06	51.11	< .001	2.85	3.08
**X1 (Group 2 vs Ref)**	-0.07	0.08	-0.87	.383	-0.22	0.09
**X2 (Group 3 vs Ref)**	0.00	0.09	0.02	.988	-0.18	0.18
**GSE**	0.17	0.10	1.68	.094	-0.03	0.37
**X1 x GSE (int_1)**	0.14	0.14	1.04	.297	-0.12	0.41
**X2 x GSE (int_2)**	-0.29	0.16	-1.84	.066	-0.60	0.02
**Constant**	2.94	0.17	17.45	< .001	2.61	3.27

**X1 (Group 2 vs Ref)**	-0.08	0.08	-0.96	.337	-0.23	0.08
**X2 (Group 3 vs Ref)**	-0.04	0.09	-0.39	.696	-0.22	0.15
**GSE**	0.12	0.10	1.21	.226	-0.08	0.33
**X1 x GSE (int_1)**	0.18	0.14	1.31	.192	-0.09	0.45
**X2 x GSE (int_2)**	-0.26	0.16	-1.63	.104	-0.57	0.05
**Organisation (covariate)**	-0.08	0.04	-1.92	.055	-0.15	0.00
**Tenure (covariate)**	0.00	0.00	-0.08	.939	-0.01	0.01
**Sex (covariate)**	0.15	0.07	2.19	.029	0.02	0.28
**Highest-order interaction test**						
**Test**	ΔR^2	F	df1, df2	p		
**X × W (RWI x GSE)**	0.01	4.10	2, 702	.017		
**X × W (RWI x GSE)**	0.01	4.16	2, 694	.016		
**Conditional effects of RWI (vs reference group) at values of GSE**						
**GSE level**	GSE value	Contrast	Effect (b)	SE	p	Estimated means (Ref / G2 / G3)
**Low (≈16th pct; −1SD)**	−0.51	X1 (G2 vs Ref)	−0.14	0.11	.181	2.88 / 2.74 / 3.03
		X2 (G3 vs Ref)	0.15	0.13	.233	
**Low (≈16th pct; −1SD)**	−0.51	X1 (G2 vs Ref)	−0.17	0.11	.115	2.92 / 2.75 / 3.01
		X2 (G3 vs Ref)	0.09	0.13	.460	
**Mean (≈50th pct)**	−0.01	X1 (G2 vs Ref)	−0.07	0.08	.373	2.97 / 2.90 / 2.97
		X2 (G3 vs Ref)	0.01	0.09	.960	
**Mean (≈50th pct)**	−0.01	X1 (G2 vs Ref)	−0.08	0.08	.327	2.98 / 2.90 / 2.94
		X2 (G3 vs Ref)	−0.03	0.09	.716	
**High (≈84th pct; +1SD)**	0.59	X1 (G2 vs Ref)	0.02	0.11	.890	3.07 / 3.08 / 2.90

		X2 (G3 vs Ref)	−0.17	0.13	.186	
**High (≈84th pct; +1SD)**	0.59	X1 (G2 vs Ref)	0.03	0.11	.796	3.05 / 3.08 / 2.86
		X2 (G3 vs Ref)	−0.19	0.13	.142	

Note: X was indicator-coded with the reference group coded as X1 = 0, X2 = 0 (Group 1: ≤3 remote days). Group 2: X1 = 1, X2 = 0; Group 3: X1 = 0, X2 = 1. GSE was mean-centered; conditional tables use the 16th, 50th, and 84th percentiles of GSE as probe values.

Robustness Check: Remote Work Intensity as a Continuous Variable

In the main text, remote work intensity (RWI) was operationalized as a three-category ordinal variable (≤3, 4–6, and ≥7 remote days in the previous 15 days). To assess whether this categorization affected the substantive conclusions, the moderation analysis was re-estimated using RWI as a continuous variable (raw count of remote workdays in the previous 15 days, range 0–15). As shown in Table S1, RWI's main effect remained non-significant for both affective rumination (AffRum) and problem-solving pondering (PSP). The RWI × GSE interaction remained significant for AffRum and non-significant for PSP, mirroring the pattern of results obtained with the categorical operationalization reported in the main text. These results indicate that the substantive conclusions of the study are not an artifact of the categorization strategy.

Table 4. Moderation Results Using RWI as a Continuous Variable (0–15 Remote Days)

**Outcome**	**Predictor**	**b**	**SE**	**p**
**AffRum**	**RWI (main effect)**	0.05	0.06	.434
**AffRum**	**RWI × GSE (interaction)**	-0.36	0.10	< .001
**PSP**	**RWI (main effect)**	-0.02	0.06	.720
**PSP**	**RWI × GSE (interaction)**	-0.14	0.11	.207

Note: b = unstandardized regression coefficient; SE = standard error. RWI = remote work intensity (continuous, 0–15 days); GSE = general self-efficacy; AffRum = Affective Rumination; PSP = Problem-solving Pondering. Models included organization, tenure, and sex as covariates.
